# Antibiotic-Resistant Bacteria in Clams—A Study on Mussels in the River Rhine

**DOI:** 10.3390/antibiotics10050571

**Published:** 2021-05-12

**Authors:** Nicole Zacharias, Iris Löckener, Sarah M. Essert, Esther Sib, Gabriele Bierbaum, Thomas Kistemann, Christiane Schreiber

**Affiliations:** 1 Institute for Hygiene and Public Health, University Hospital Bonn, Medical Faculty University of Bonn, Venusberg-Campus 1, 53127 Bonn, Germany; sarah.essert@ukbonn.de (S.M.E.); esther.sib@ukbonn.de (E.S.); thomas.kistemann@ukbonn.de (T.K.); christiane.schreiber@ukbonn.de (C.S.); 2 Institute for Pharmaceutical Microbiology, University Hospital Bonn, Meckenheimer Allee 168, 53115 Bonn, Germany; iris.loeckener@uni-bonn.de; 3 Institute of Immunology, Medical Microbiology and Parasitology, University Hospital Bonn, Medical Faculty University of Bonn, Venusberg-Campus 1, 53127 Bonn, Germany; gabi.bierbaum@ukbonn.de; 4 Department of Geography, University of Bonn, Meckenheimer Allee 166, 53115 Bonn, Germany

**Keywords:** antibiotic resistances, environmental health, food chain, infection risk, multidrug resistance, shellfish

## Abstract

Bacterial infections have been treated effectively by antibiotics since the discovery of penicillin in 1928. A worldwide increase in the use of antibiotics led to the emergence of antibiotic resistant strains in almost all bacterial pathogens, which complicates the treatment of infectious diseases. Antibiotic-resistant bacteria play an important role in increasing the risk associated with the usage of surface waters (e.g., irrigation, recreation) and the spread of the resistance genes. Many studies show that important pathogenic antibiotic-resistant bacteria can enter the environment by the discharge of sewage treatment plants and combined sewage overflow events. Mussels have successfully been used as bio-indicators of heavy metals, chemicals and parasites; they may also be efficient bio-indicators for viruses and bacteria. In this study an influence of the discharge of a sewage treatment plant could be shown in regard to the presence of *E. coli* in higher concentrations in the mussels downstream the treatment plant. Antibiotic-resistant bacteria, resistant against one or two classes of antibiotics and relevance for human health could be detected in the mussels at different sampling sites of the river Rhine. No multidrug-resistant bacteria could be isolated from the mussels, although they were found in samples of the surrounding water body.

## 1. Introduction

### 1.1. Antibiotic-Resistant Bacteria in Water Systems

Numerous bacterial species occur naturally in surface water bodies, but pathogenic antibiotic-resistant bacteria (ARB) and antibiotic resistance genes (ARGs) can also be found [1,2,3,4]. Faecal pathogenic bacteria, including ARB and ARGs, enter surface water bodies through various pathways, i.e., point sources such as effluents of sewage treatment plants (STPs) and combined sewer overflows [5,6,7], as well as diffuse surface or subsurface run-off from non-sealed landscapes [8]. Wastewater from health care facilities, such as hospitals or nursing homes, and the agro-food industry are further known sources of human-pathogenic and/or (multi-)resistant bacteria and various types of resistances [9,10]. Antibiotics excreted into wastewater (WW) are an additional reason for the spread of antibiotic resistance [11].

Acquired resistance genes are often located on plasmids and can be passed onto other bacteria via horizontal gene transfer. In this process, WW and STPs are of particular importance due to the high density of bacteria [12]. The WHO classified ARB as a serious threat to modern medicine [13] and categorized specific organisms based on their clinical significance and resistance capabilities into three priority classes. Carbapenem-resistant *Acinetobacter baumannii*, *Pseudomonas aeruginosa* and Enterobacterales were thereby grouped as the first priority (“critical”) whereas vancomycin-resistant *Enterococcus faecium* (VRE) and methicillin-resistant *Staphylococcus aureus* (MRSA) are in the second priority group (“high”) [14].

### 1.2. Mussel Species in the River Rhine

The river Rhine is a large, well-monitored, cross-border-river that receives substantial amounts of treated wastewater. It originates in the Swiss canton of Grisons and flows through Germany and the Netherlands into the North Sea. Besides a large and diverse flora, the river Rhine also contains a variety of fish and small animal species such as insects, snails and mussels. Endemic mussels include the duck mussel (*Anodonta anatina*), the painter’s mussel (*Unio pictorum*) and the common river mussel (*Unio crassus*) [15]. The river Rhine is also populated by invasive mussel species, such as *Corbicula* spp. [16] and *Dreissena* spp. [17]. Both species are so numerous that they account for the majority of current shellfish in the river Rhine.

*Corbicula* spp. occur in large populations and are a popular food source [18]. They were first detected in European surface waters in 1980 [19] and spread throughout connected water and river systems. Mussels of the genus *Corbicula* occurring in the river Rhine are *C. fluminea* and *C. fluminalis*.

Of the genus *Dreissena,* the species *D. polymorpha* and *D. rostriformis bugensis* are of interest in the river Rhine. They vary widely in shell shape, colour and stripe pattern on the shell [20]. *Dreissena* spp. have byssus threads, which they use to attach themselves to stones, wood, other large mussels, metal or other solid surfaces [21,22]. They are sessile, but can dissolve the byssus threads by enzymes. Both mentioned species occur in rivers, lakes and harbours, but are most common in canals [22]. *D. polymorpha* was discovered for the first time in the Upper Rhine attached to a raft in the Mannheim harbour in 1840 [17]. The species *D. rostriformis bugensis* was first detected much later in an estuary of the river Rhine in the Netherlands (Hollands Diep) in 2006 [23]. From there, it migrated upstream and was found in 2008 in the Lower Rhine region between Dormagen and Bimmen [24].

Mussels use suspension filtration to acquire nutrients. Water is taken up into the interior of the shell through the siphon and phytoplankton, microorganisms and organic material are filtered out [25,26,27]. Mussels are established bio-indicators for heavy metals, chemicals and parasites but are also considered for viruses and bacteria [28,29]. They can accumulate large quantities of microorganisms from their surrounding waters, including opportunistic bacteria (*Aeromonas*, *Vibrio*, *Pseudomonas*), protozoan parasites (*Cryptosporidium*, *Giardia*), viruses (adenoviruses, hepatoviruses) and pathogenic bacteria (*Escherichia coli*, *Salmonella*) [30]. They may therefore jeopardize human health, particularly when consumed as seafood [31]. Both mussel genera investigated in this study are edible [18], albeit the ingestion of *D. polymorpha* is not recommended due to the proven transmission of the parasites *Toxoplasma gondii*, *Giardia* spp. and *Cryptosporidium* spp. [32,33].

Mussels living in surface waters that contain pathogenic bacteria and ARB may represent a reservoir of those microorganisms, and thus can be used as indicators for their presence in a water environment.

## 2. Results

In total, 22 mussels were obtained from three different sampling sites (Table 1). Nine mussels belonging to *Corbicula* spp. were harvested upstream the municipal STP of Bonn and eight mussels belonging to the same species were selected downstream of the municipal STP. Five mussels of *Dreissena* spp. could be harvested from the sampling location in the river in Cologne. The *Corbicula* spp. sampled upstream of the municipal STP had an average tissue weight of 1.4 g, whereas mussels found downstream the municipal STP showed an average weight of 0.4 g. The *Dreissena* spp. soft mussel tissue weighted 0.2 g each. Water temperatures over the study period ranged between 22.0 °C and 28.3 °C.

### 2.1. Bacterial Flora in the Mussel Tissue

**Figure 1:**
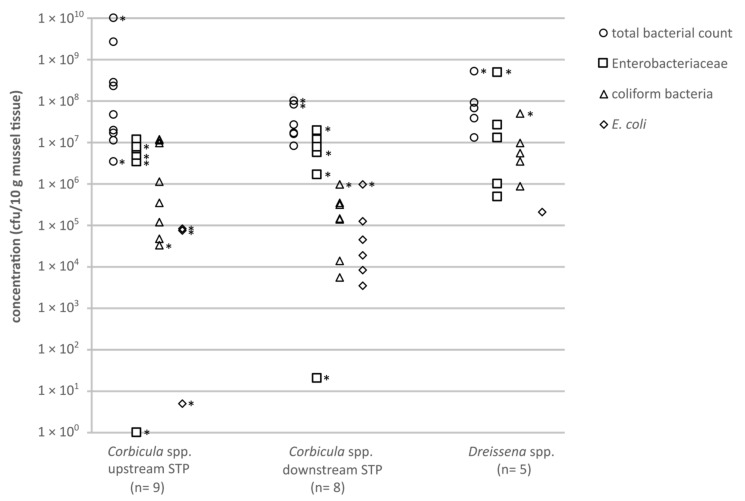
Concentrations (cfu/10 g mussel soft tissue) of clinically relevant bacteria from mussels, cultivated on different agars: circles = total bacteria count (TBC; Columbia blood agar); squares = Enterobacteriaceae (MacConkey agar); triangles = coliform bacteria (CC agar); diamonds = *E. coli* (CC agar). Asterisks mark category values of ≥x cfu/10 g mussel tissue, the smallest possible value is shown.

The abundance of potentially clinically relevant pathogens in the mussels was investigated by using different agar plates. The total bacterial counts (TBCs) (Columbia blood agar), Gram-negative bacteria (MacConkey agar) and coliform bacteria (chromocult coliform (CC) agar), as well as *E. coli* (CC agar) were determined to evaluate the bacterial flora of the two mussel species and a potential influence of WW and the STP. The bacterial abundance was calculated in colony forming units per 10 g of mussel soft tissue to enable comparison of the samples (Figure 1).

The TBCs of bacteria in the mussel soft tissue of *Corbicula* spp. were diverse. Mussels sampled upstream of the municipal STP in Bonn showed results between 3.4 × 10^6^ and 1.0 × 10^10^ cfu/10 g (Figure 1). In mussels sampled downstream of the municipal STP, the TBCs ranged between 8.0 × 10^6^ and 1.0 × 10^8^ cfu/10 g. The TBCs on Columbia blood agar detected for the genus *Corbicula* were slightly higher upstream compared to downstream of the municipal STP.

The concentrations of Gram-negative bacteria, in the mussels *Corbicula* spp. ranged from 1 up to >2.0 × 10^7^ cfu/10 g upstream the STP and 20 up to >2.0 × 10^7^ cfu/10 g downstream the STP. The bacterial concentrations were below the individual limit of detection (LOD_i_) for two mussels samples (<43 and <48 cfu/10 g). Varying detection limits resulted from the different weights of the sampled mussel soft tissue (Table 1).

The concentrations of coliform bacteria in *Corbicula* spp. ranged between 3.4 × 10^4^ and 1.2 × 10^7^ cfu/10 g upstream the STP. Downstream the STP, concentrations of 5.5 × 10^3^ up to >1.0 × 10^6^ cfu/10 g were detected. In five out of nine samples upstream the STP, the number of *E. coli* in *Corbicula* spp. soft tissue was below the LOD_i_. The maximum concentration was >8.3 × 10^4^ cfu/ 10 g. The concentration of *E. coli* in the mussels *Corbicula* spp. found downstream the local STP was below the LOD_i_, with a maximum of >1 × 10^6^ cfu/10 g. Results <LOD_i_ are not shown in the figure.

For mussel species of the genus *Dreissena* (D1–D5) the concentration of bacteria grown on Columbia blood agar ranged between 1.3 × 10^7^ and >5.0 × 10^8^ cfu/10 g. The concentrations for the Gram-negative bacteria and coliform bacteria varied between 5.0 × 10^5^ and >5.0 × 10^6^ cfu/10 g and 9.0 × 10^5^ and >5.0 × 10^7^ cfu/10 g, respectively. In four out of five samples, *E. coli* could not be detected.

### 2.2. Distribution of Antibiotic-Resistant Bacteria

**Figure 2:**
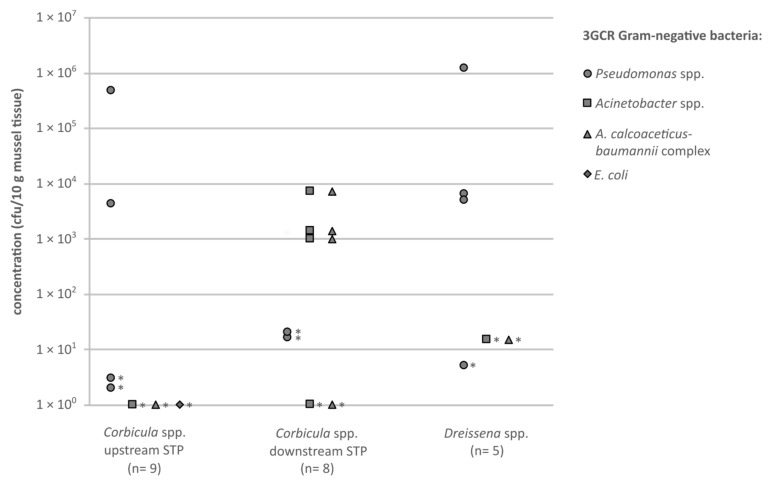
Concentrations (cfu/10 g mussel soft tissue) of antibiotic-resistant (3GCR) Gram-negative bacteria, cultivated on CHROMagar ESBL (circles = *Pseudomonas* spp.; squares = *Acinetobacter* spp.; triangles = *A. calcoaceticus-baumannii* complex; diamonds = *E. coli*), originating from the tissue of the investigated mussels. Asterisks mark category values of ≥x cfu/10 g mussel tissue, the smallest possible value is shown.

Methicillin-resistant *S. aureus*, vancomycin-resistant *E. faecium* and *E. faecalis*, extended spectrum beta-lactamase (ESBL)-producing *Klebsiella* spp., *Enterobacter* spp. and *Citrobacter* spp. (grouped as KEC) and ESBL-producing *Proteus* spp. could not be detected in neither the sampled *Corbicula* spp. nor the *Dreissena* spp. mussels. The numbers of colonies of these bacterial species were below the individual detection limits, which varied between <36 and <1000 cfu/10 g depending on the weight of the mussel tissue. Only one isolate of an ESBL-producing *E. coli* was obtained from a mussel of the *Corbicula* spp. upstream the STP. *Pseudomonas* spp., resistant against third-generation cephalosporins (3GCR), were detected frequently in all mussel species and sampling sites; none of these were *P. aeruginosa* 3GCR (Figure 2). All resistant *Acinetobacter* spp. detected belonged to the *A. calcoaceticus-baumannii* complex. Results <LOD_i_ are not shown in the figure. Each isolate was confirmed by matrix-assisted laser desorption/ionisation time of flight mass spectrometry (MALDI-TOF-MS) and tested for its susceptibility against the antibiotic substances shown in Table 2.

**Figure 3:**
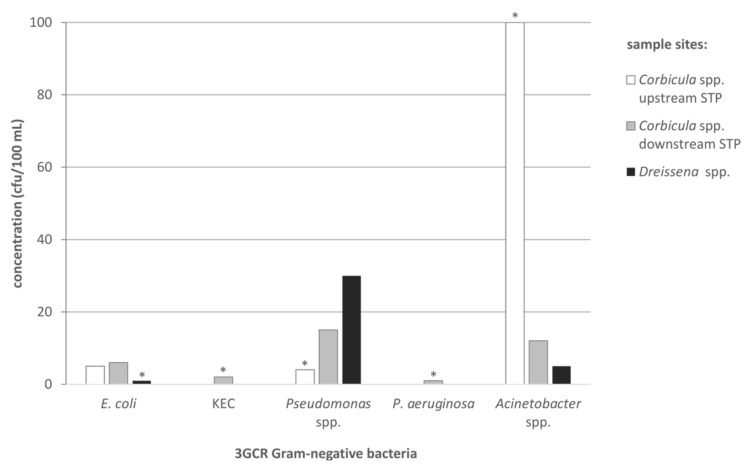
Concentrations (cfu/100 mL) of antibiotic-resistant Gram-negative bacteria (*E. coli*, KEC, *Pseudomonas* spp., *P. aeruginosa*, *Acinetobacter* spp.), cultivated on ESBL CHROMagar, originated from the water of the investigated sampling sites (white bars = *Corbicula* spp. upstream of the STP; grey bars = *Corbicula* spp. downstream of the STP; black bars = *Dreissena* spp.) during the time of mussel harvesting. Asterisks mark category values of ≥x cfu/100 mL river water, the smallest possible value is shown.

MRSA, VRE and ESBL-producing *Proteus* spp. could not be detected in any water sample consisting of 100 mL river water from the mussel sampling sites. ESBL-producing *E. coli* and KEC were detected in low concentrations from 1 up to 6 cfu/100 mL and ≥2 cfu/100 mL in one sample, respectively (Figure 3). These two KEC isolates were identified by MALDI-TOF-MS as *K. pneumoniae*. Both isolates were detected in the sampled water downstream of the local STP. *Pseudomonas* spp. 3GCR, could be detected in all samples at different concentrations (≥4–30 cfu/100 mL); but only one isolate of *P. aeruginosa* 3GCR could be obtained from a water sample downstream of the municipal STP. *Acinetobacter* spp. 3GCR were present in all water samples ranging from 5 to >100 cfu/100 mL), all belonging to the *A. calcoaceticus*-*baumannii* complex.

The isolates were also investigated for their individual resistance profiles. All bacterial isolates from CHROMagar ESBL found in mussel soft tissue and water showed resistance to the broad-spectrum cephalosporin cefotaxime and 28.6% additionally against ceftazidime (Table 2). Also, 14.3% of the isolates showed phenotypic resistance against ciprofloxacin.

Isolates with a multidrug resistance status originated from river water. One isolate belonging to the *A. calcoaceticus-baumannii* complex and one *E. coli* isolate had a 3MRGN status and one *P. aeruginosa* (3GCR) isolate was classified as 4MRGN. Further investigation showed that this *P. aeruginosa* strain is a member of ST316 and harboured the carbapenemase gene *bla*VIM. Most ARB detected in the mussels belong to the *A. calcoaceticus-baumannii* complex and one isolate could be identified as ESBL-producing *E. coli*. The latter was a single isolate gained from a *Corbicula* spp. mussel sampled upstream the municipal STP (Cu). This isolate showed phenotypic resistance against the cephalosporins cefotaxime and ceftazidime as well as against the fluoroquinolone ciprofloxacin. However, it was still susceptible to the ureidopenicillin piperacillin in combination with tazobactam, as well as the carbapenems imipenem and meropenem. No extensively drug resistant (XRD) strains could be isolated from the mussel tissue or the river water.

## 3. Discussion

Ensuring that the examined mussels were vital, the environmental factor temperature was evaluated. According to a previous study, mussels of the species *Corbicula* spp. and *Dreissena* spp. can tolerate water temperatures of 2 to 36 °C [34]. The average water temperature of the river Rhine during the sampling period varied from 22.0 to 28.3 °C. These temperatures are in the normal range of the living conditions for both genera of mussels.

### 3.1. Influence of Treated Wastewater on the Bacterial Flora in River Mussels

The concentrations of potential clinically relevant bacteria were determined in the mussel soft tissue of each mussel species at each sampling site to analyse the influence of various sampling parameters. Cultivated isolates on Colombia blood agar, Gram-negative bacteria cultivated on MacConkey agar, as well as coliform bacteria and *E. coli* cultivated on CC agar were included in the investigation. This study presents an orientational investigation on the occurrence of antibiotic resistant bacteria in mussels. Although it is based on a limited number of samples, some valuable insights can be gained.

Mussels are known to be able to accumulate heavy metals in their tissue and shell, showing a high tolerance to the effects of these metals [35]. As suspension filter feeders, they have high filtration rates [25,26,27]. Bighiu et al. (2019) [36] demonstrated in lab scale experiments that mussels also accumulate bacteria. The concentration of the indicator bacteria *E. coli* and enterococci were 132 times higher compared to the respective water sample, when sewage effluent had been used to feed the mussels (mussel tissue and shell were analysed). They also showed that bacteria can persist up to 48 h in the mussels before digestion and that peak exposures with faecal bacteria by water contamination can be monitored by investigating mussels [36].

The bacterial flora within the mussel tissue of *Corbicula* spp. in this study was diverse and relatively constant across sampling sites, except for the more frequent detection of *E. coli* downstream of the municipal STP. The presence of the faecal indicator *E. coli* can be linked to the influence of WW at the sampling site. Even though the individual LOD_i_ for the mussels upstream the STP was lower due to their higher weight, *E. coli* could only be detected in four out of nine samples. Araujo et al. (1993) described a high variety in the sizes of adults of *Corbicula* spp. Small mussels seem to be more affected by the composition of their habitat, as they use amino acids as an energy source; larger mussels primarily draw on their larger storage reserves of carbohydrates [37]. The effect by chemical components in the water on the growth rate of *Corbicula* spp. could already be shown by Belanger et al. (1990) [38]. Whether the size variations of the mussels stemmed from a natural variation or were due to the water quality at the sampling sites could not be finally evaluated by this study setting. Nevertheless, all sampled mussels downstream the STP were smaller, pointing to an effect of water quality on mussel growth.

### 3.2. Antibiotic-Resistant Bacteria in the Mussel Tissue

Bighiu et al. (2019) [36] found multidrug resistant bacteria at 2–5 times higher concentrations in the mussels (shell and tissue) than in the water sample. The water samples analysed in this study show relatively low concentrations of ARB except for bacteria from the *A. calcoaceticus-baumannii* complex, which could be found in concentrations >100 cfu/100 mL river water (sample upstream the STP). The selected ARB were not detected in six of seventeen *Corbicula* spp. mussels examined, of which four out of nine were collected upstream of the STP and two out of eight collected downstream of the STP. Four out of five mussels of the genus *Dreissena* were tested positive for at least one of the investigated ARB.

In most of the mussel tissue samples (73%) of *Corbicula* spp. and *Dreissena* spp., ESBL-producing *E. coli,* bacteria belonging to the *A. calcoaceticus-baumannii* complex or *Pseudomonas* spp. resistant against third-generation cephalosporins were detected. These resistant bacteria seem to be relatively persistent within mussels. However, ESBL-producing *E. coli* in mussels was a single isolate gained from a sample of a *Corbicula* spp. upstream of the STP, and VRE were not detected within the mussel tissue. This is in accordance with the limited persistence of faecal bacteria in aquatic environments. In water samples, VRE were not detected whereas ESBL-producing *E. coli* could be extracted in low concentrations. The 4MRGN *P. aeruginosa* strain, isolated from the river water downstream the STP, harboured the carbapenemase gene *bla*VIM. As a member of ST316, it does not belong to known high risk strains and has been mainly reported in Asia [39,40,41]. In case of a positive result for ARB in the mussel tissue, the concentrations varied within 3 log10 cfu/10 g. None of the isolates belonged to the XDR group confirming results published by Müller et al. (2018) [42].

In addition to studies which used mussels as bio-indicators for faecal contamination, various studies investigated the ability of *C. fluminea* and *D. polymorpha* to remove *E. coli* from water [25,43]. It was demonstrated that *D. polymorpha* can digest and metabolise 100% of the bacteria and thus remove them irreversibly from the water body [43]. In *C. fluminea*, the removal rate was up to 98%, most likely because of the slightly lower filtration rate of 33 mL × g^−1^ × h^−1^ [44] compared to 5–400 mL × g^−1^ × h^−1^ in *D. polymorpha* [25]. These results suggest that both mussel species can filter and digest bacteria from water, so that overall, a smaller diversity of bacterial species was present as shown in the present study.

The detected ARB probably originated from the mucus of the mussels, clung to the surface of the mussel tissue or had not yet been digested. Although the water quality and ecology of the river Rhine have been improved over the last decades, e.g., through optimization and modernization of STPs supported by international programmes like “Rhine 2020—Program on the sustainable development of the Rhine” [45], the wastewater discharges into the river Rhine still emit substantial amounts of resistant ESBL-producing Gram-negative bacteria [9]. Our study demonstrates the persistence of resistant human pathogens in the river water and in mussels. Following the water course, such bacteria may spread unnoticed, posing risks to human health through various pathways. Multiple studies have shown that the environment, including water bodies like rivers, is affected by the spread of antibiotic resistance [46]. More multidrug resistant isolates were detected in water than in mussels. However, when present, the concentrations of detected ARB were higher in the mussels than in the corresponding water sample.

## 4. Materials and Methods

### 4.1. Sampling Sites

*Corbicula* spp. was sampled at two sampling sites located on the right bank of the river Rhine near the city of Bonn, Germany. The study was designed to include sampling sites with and without the influence of treated WW. The first site was upstream of the municipal STP and not influenced by its discharges, as there is no further STP for at least 7.5 km upstream of the sampling site (*Corbicula* spp. upstream STP). The second site was located on the same river bank but approx. 500 m downstream of the outlet of the STP (which is located on the left river side and connected to various hospitals and one maximum care hospital) (*Corbicula* spp. downstream STP). Thus, this site was directly influenced by treated WW. All sites used for harvesting of *Corbicula* spp. were reachable from the banks of the river Rhine and no more than 2 m away from the shore; the water level varied between approximately 0.5 and 1.0 m.

Mussels of the genus *Dreissena* were sampled at one sampling site located on the left bank of the river Rhine in Cologne, Germany. The mussels were collected from stones below the grid of the Ecological Rhine station of the University of Cologne. Mussels of the genus *Dreissena* were collected 1 to 4 m from the shore at a water depth of no more than 0.3 m. A STP is located 3.4 km upstream of this sampling site, which processes WW from urban areas with no connection to hospitals.

### 4.2. Sampling Procedure

The mussels were taken from their natural habitat, resulting in different sampling schemes. *Corbicula* spp. which were buried in the sand could be found at the water edge. They were harvested with a shovel or a sample beaker (tied to a telescopic pole) and dug out of the sand. Mussels of the genus *Dreissena* were picked from stones, detached *Dreissena* spp. were not examined. To loosen mussels of *Dreissena* spp. from the substrate, the byssus threads were severed with a knife or carefully detached by hand.

Only living and healthy mussels were analysed. Vitality was tested by gently trying to pull both halves of the shell apart. If this was not successful, it could be assumed that the mussels were in a vital condition. The mussels were separated according to type and location and transferred in sterile bottles that were filled with the water from the respective sampling location to keep the mussels alive until examination in the laboratory. In addition to the analysis of the mussels, water samples were taken for each sampling site and sampling campaign. For this, 100 mL of water were taken five times at 2-min intervals to generate mixed samples according to the German standard method for the examination of water, wastewater and sludge [47].

To further ensure that the examined mussels were vital and thus undergoing suspension filtration, the temperature was evaluated. All samples were cooled during the transport to the laboratory and kept at 2–8 °C until being processed within the next 24 h.

### 4.3. Preparation of Mussel Samples

All following steps were carried out under sterile conditions: The mussels were individually transferred into a petri dish each and measured in width and length. The byssus threads of *Dreissena* spp. were removed using a scalpel and discarded. To open the shells, the scalpel was inserted into the lateral inflow or outflow channels and passed along the shell until both halves of the shell could be pulled apart. The anterior and posterior shell adductors and retractors were detached with the scalpel and the mantle was removed from the hypostracum, using tweezers. The soft tissue was weighed, transferred to a sterile 50 mL sampling container that contained 10 mL of 0.9% (*w*/*v*) NaCl and crushed until a homogeneous suspension was obtained.

### 4.4. Determination of the Relevant Bacterial Flora in Mussels

Enabling the inclusion of different bacterial species, the mussel tissue samples were cultivated using different nutrient media. The TBC was performed using Columbia blood agar (Oxoid, Wesel, Germany) after an incubation at 37 °C for 24 h. *E. coli* and coliform bacteria were analysed by using CC agar (Merck, Darmstadt, Germany) supplemented with a selective supplement (Merck; 2.5 mg vancomycin and 2.5 mg cefsulodine per 500 mL media) to inhibit the growth of *Pseudomonas* spp., *Aeromonas* spp. and Gram-positive bacteria; incubation was performed at 37 °C for 24 h. Gram-negative bacteria were determined using MacConkey agar (Oxoid, Wesel, Germany) after an incubation at 37 °C for 24 h. The homogenised mussel sample was diluted and spread onto the culture media. For each sample, if possible, two to three dilution steps were tested to obtain at least one evaluable dilution. After incubation on blood agar and MacConkey agar plates, the TBC was determined by counting all colonies, independent of appearance. For the CC agar, the colonies were counted separately by colour, according to the manufacturer’s instructions. While *E. coli* grows in dark blue to violet colonies, *Enterobacter aerogenes*, *Citrobacter freundii* and other coliforms form pink to red colonies. The resulting bacterial concentrations were calculated according to ISO 8199 [48].

### 4.5. Detection of Antibiotic-Resistant Bacteria

The water samples and the mussel cell suspensions were tested for the presence of antibiotic-resistant bacteria using chromogenic agar plates (CHROMagar ESBL, CHROMagar MRSA and CHROMagar VRE; MAST Diagnostica, Reinfeld, Germany), which are normally used for the isolation and differentiation of ARB from human material. A detailed description with performance characteristics for environmental samples was recently published by Schreiber et al. (2021) [49]. Depending on the expected target bacteria and background flora, 1 mL and/or a dilution of the sample was spread directly on agar plates. Volumes > 1 mL up to 100 mL were filtered through a cellulose nitrate filter (mixed cellulose ester, diameter 50 mm, pore size 45 µm). The plates were then incubated for 24 h at 42 °C for CHROMagar MRSA and CHROMagar ESBL and for 48 h at 42 °C for CHROMagar VRE. Of those media, nine different bacterial species were selected as target organisms, considering the priority list of ARB to guide research, discovery and development of new antibiotics [14]. Microbiological parameters included clinically relevant Gram-negative bacteria and resistant phenotypes: ESBL-producing *Klebsiella* spp., *Enterobacter* spp., *Citrobacter* spp. (grouped as “KEC”, due to morphological similarity on the CHROMagar ESBL plates), *E. coli*, *Proteus mirabilis* as well as *P. aeruginosa* and species belonging to the *Acinetobacter calcoaceticus-baumannii* complex showing resistance to 3rd-generation cephalosporins (3GCR). As Gram-positive clinically relevant resistant bacteria the following species were selected: methicillin-resistant *S. aureus* (MRSA) and the vancomycin-resistant enterococci (VRE) *E. faecium* and *E. faecalis*.

Confirmation and identification of bacterial species were performed using matrix-assisted laser desorption/ionisation time of flight mass spectrometry (MALDI-TOF MS) and with a VITEK^®^ MS mass spectrometer (bioMérieux, Marcy l’Etoile, France), employing the Myla^TM^ software.

For identification of the resistance profiles, bacteria belonging to the *A. calcoaceticus-baumannii* complex, Enterobacterales and *P. aeruginosa* were tested for antibiotic resistance using the microdilution assay Micronaut-S MDR MRGN-Screening 3 system (MERLIN, Bornheim-Hersel, Germany). The interpretation of the susceptibility status was performed according to the European Committee on Antimicrobial Susceptibility Testing criteria (EUCAST, Version 9.0, 2019).

With regard to multidrug resistance, all Gram-negative isolates were tested for their susceptibility against three of four clinically relevant antibiotic classes (piperacillin/tazobactam, fluoroquinolones (ciprofloxacin), third-generation cephalosporins (cefotaxime and/or ceftazidime) and carbapenems (meropenem and/or imipenem)) were classified as 3MRGN (multidrug resistant Gram-negative bacteria). All isolates that showed resistance to all four antibiotic groups mentioned above were classified as 4MRGN. The use of piperacillin/tazobactam is a modification of the test standards defined by the German Commission for Hospital Hygiene and Infection Prevention (KRINKO), which includes solely piperacillin. However, the latter is not used as a single drug in clinical therapy in Germany [50,51]. Therefore, we tested the commonly used combination with tazobactam. In case of the detection of a carbapenemase gene, the isolate was deemed 4MRGN independently of the phenotypical resistance against the tested antibiotics.

### 4.6. Molecular Typing of Resistant Isolates

For multilocus sequence typing (MLST) of multidrug resistant isolates (4MRGN), two to three colonies from fresh Columbia agar plates were incubated for 5 min at 95 °C in 80 μL of ultra purewater. The suspension was spun down at 14,000× *g* and the PCR was conducted according to previously described DLST standard procedures for *P. aeruginosa* [39,40]. The PCR products were purified from 1% agarose gels using the GeneJET Gel Extraction Kit (Fisher Scientific GmbH, Schwerte, Germany) and sequenced by GATC Biotech AG (Konstanz, Germany). Sequence types were determined via the specific MLST website for *P. aeruginosa* (https://pubmlst.org/organisms/pseudomonas-aeruginosa/) (accessed on 15 March 2021) [52].

### 4.7. Determination of Individual Limit of Detection

An individual limit of detection (LOD_i_) occurred depending on the weight of the mussel and the initial expected bacteria concentration and background flora. The general LOD_i_ can be calculated by using the volume (10 mL) divided by soft tissue weight. For the very small mussels (0.1 g soft tissue) the LOD_i_ was 100 cfu/10 g or even 1000 cfu/10 g, in those cases that only 10 mL of the soft tissue suspension could be analysed and was found negative for the target species.

### 4.8. Graphical Representation of the Results

The data are presented as individual data points (Figure 1 and Figure 2) and as bars in Figure 3 (single values are shown). The limitation of the microbiological analysis, caused majorly by accompanying background flora, leads to results that indicate a minimum value and no exact value. In the case of results that indicate values ≥x cfu/10 g for the mussel tissue or ≥x cfu/100 mL for the water samples, the smallest possible value is entered in the graph. Such a value is marked with an asterisk in the respective figures.

## 5. Conclusions

The presence of faecal bacteria in the mussels points to an impact of treated wastewater on the individual mussel. Antibiotic-resistant isolates could be detected in the soft tissue of mussels of the genera *Dreissena* and *Corbicula* from different sampling sites of the river Rhine. No multidrug-resistant bacteria could be isolated from the mussel tissue, but were found in samples of the corresponding river water. In accordance with other work, a higher number of antibiotic-resistant bacteria (not multidrug-resistant) could be detected in the mussels as compared to the water. The diversity of antibiotic-resistant bacteria was higher in the water samples, reflected by different persistence. The cleaning performance of mussels naturally inhabiting river systems on the microbiological water quality cannot be estimated by these results and needs to be examined in further studies. The presence of antibiotic-resistant Gram-negative bacteria in the river Rhine and within mussels indicates that some resistance genes are widespread or even ubiquitous in water courses.

## Figures and Tables

**Table 1:** Individual mussel soft tissue weight measured without shells and sampled at the three different sampling sites.

Mussel Species Investigated in the River Rhine														
	*Corbicula* spp. upstream STP (Cu)									*Dreissena* spp. (D)				
	Cu1	Cu2	Cu3	Cu4	Cu5	Cu6	Cu7	Cu8	Cu9	D1	D2	D3	D4	D5
weight mussel tissue (g)	0.6	0.4	2.3	1.0	2.8	2.1	1.3	1.2	1.3	0.2	0.2	0.2	0.2	0.2
	*Corbicula* spp. downstream STP (Cd)													
	Cd1	Cd2	Cd3	Cd4	Cd5	Cd6	Cd7	Cd8						
weight mussel tissue (g)	1.2	0.3	0.4	0.4	0.1	0.1	0.1	0.6						

**Table 2:** Antibiotic-resistant isolates of the bacteria (isolated on the CHROMagar ESBL plates) from different sampling sites and matrices, detection of carbapenemase genes within the isolates and classification of German (3/4MRGN = multi-resistant Gram-negatives with resistance against three or four antibiotic groups tested) and international (XDR = extensively drug-resistant; showing resistance against all but one or two reserve antibiotic classes) multidrug resistance levels.

		Antibiotic Substances									
	Species (Number of Isolates)	Piperacillin/ Tazobactam	Cefotaxime	Ceftazidime	Imipenem	Meropenem	Ciprofloxacin	Carbapenemase	3MRGN	4MRGN	XDR
*Corbicula* spp. (upstream STP)	*E. coli* (1)	0	1 (100%)	1 (100%)	0	0	1 (100%)	0	0	0	0
	*A. calcoaceticus-baumannii* complex (1)	0	1 (100%)	0	0	0	0	0	0	0	0
River water Bonn (upstream STP)	*E. coli* (4)	0	4 (100%)	3 (75%)	0	0	0	0	0	0	0
	*A. calcoaceticus-baumannii* complex (6)	1 (17%)	6 (100%)	0	0	0	0	0	0	0	0
*Corbicula* spp. (downstream STP)	*A. calcoaceticus-baumannii* complex (8)	0	8 (100%)	2 (25%)	0	0	1 (13%)	0	0	0	0
River water Bonn (downstream STP)	*E. coli* (2)	1 (50%)	2 (100%)	2 (100%)	0	0	1 (50%)	0	1 (50%)	0	0
	*K. pneumoniae* (2)	0	2 (100%)	1 (50%)	0	0	1 (50%)	0	0	0	0
	*P. aeruginosa* (1)	0	1 (100%)	1 (100%)	0	1 (100%)	0	1 (100%)	0	1 (100%)	0
	*A. calcoaceticus-baumannii* complex (3)	0	3 (100%	0	0	0	0	0	0	0	0
*Dreissena* spp.	*A. calcoaceticus-baumannii complex* (2)	0	2 (100%)	0	0	0	0	0	0	0	0
River water Cologne	*E. coli* (1)	0	1 (100%)	0	0	0	1 (100%)	0	0	0	0
	*A. calcoaceticus-baumannii* complex (4)	1 (25%)	4 (100%)	0	0	0	0	0	1 (25%)	0	0
Isolates total	35	3 (8.6%)	35 (100%)	10 (28.6%)	0	1 (0.3%)	5 (14.3%)	1 (2.9%)	2 (5.7%)	1 (2.9%)	0

## Data Availability

All data supporting the results are shown in this study.
